# Breast Desmoid-Type Fibromatosis Mimicking Carcinoma on Imaging: A Case Report

**DOI:** 10.70352/scrj.cr.26-0213

**Published:** 2026-06-13

**Authors:** Mizuki Mizuuchi, Kayono Onishi, Yoshiya Horimoto, Yoichi Koyama, Kyoko Orimoto, Natsuki Uenaka, Hiroki Kusama, Takeshi Ushigusa, Maki Tanigawa, Eiichi Sato, Noriko Kobayashi, Takashi Ishikawa

**Affiliations:** 1 Department of Breast Surgery and Oncology, Tokyo Medical University, Tokyo, Japan; 2 Department of Anatomic Pathology, Tokyo Medical University, Tokyo, Japan; 3 Department of Pathology (Medical Research Center), Institute of Medical Science, Tokyo Medical University, Tokyo, Japan; 4 Department of Radiology, Tokyo Medical University, Tokyo, Japan

**Keywords:** desmoid-type fibromatosis, breast, spiculated mass, breast imaging

## Abstract

**INTRODUCTION:**

Desmoid-type fibromatosis of the breast is a rare, locally aggressive fibroblastic tumor that lacks metastatic potential but demonstrates infiltrative growth and a tendency for local recurrence. Because its imaging characteristics often resemble those of breast carcinoma, establishing a preoperative diagnosis can be challenging.

**CASE PRESENTATION:**

A 57-year-old woman was referred after screening mammography detected a spiculated, high-density mass in the right breast. Ultrasonography demonstrated an irregular hypoechoic mass measuring approximately 36 mm in the upper inner portion of the breast with peripheral vascularity, findings suspicious for malignancy. Core needle biopsy revealed proliferation of spindle-shaped cells with minimal atypia and nuclear β-catenin positivity, suggesting desmoid-type fibromatosis. Tumor excision was subsequently performed for definitive diagnosis and local control. Histopathological examination confirmed desmoid-type fibromatosis with infiltrative growth into the surrounding adipose tissue. Focal margin involvement was identified, and the patient has been managed with careful follow-up.

**CONCLUSIONS:**

Breast desmoid-type fibromatosis is a rare tumor that can closely mimic breast carcinoma on imaging studies. Histopathological evaluation is essential for establishing the diagnosis. Because positive surgical margins have been associated with an increased risk of local recurrence, careful postoperative follow-up is warranted.

## INTRODUCTION

Desmoid-type fibromatosis is a rare soft tissue tumor characterized by the proliferation of fibroblasts and myofibroblasts. In the current WHO Classification of soft tissue and bone tumours, it is categorized as an intermediate, locally aggressive neoplasm that demonstrates local infiltrative growth without distant metastasis but has a high rate of local recurrence.^1)^ In the Japanese Breast Cancer Society’s General Rules for Clinical and Pathological Recording of Breast Cancer, it is classified as an intermediate tumor within the category of soft tissue tumors.^2)^ Primary involvement of the breast is extremely rare, accounting for approximately 0.2%–0.3% of all breast tumors.^3)^ Although this disease can arise in virtually any anatomical site, primary involvement of the breast represents a subtype of extra-abdominal desmoid and is extremely rare. On imaging, it often presents as an irregular mass or an ill-defined lesion, which may make differentiation from breast carcinoma challenging.^4,5)^

We herein report a case of primary breast desmoid-type fibromatosis that was initially suspected to be breast cancer on screening mammography and ultrasonography, suggested by repeated core needle biopsies, and treated with tumor excision, together with a review of the relevant literature.

## CASE PRESENTATION

A 57-year-old woman was referred to our hospital for further evaluation and treatment after screening mammography revealed a high-density mass with spiculated margins in the right breast. She had a history of diabetes mellitus and hypertension. She had no history of breast augmentation, prior breast surgery, or trauma. There was no remarkable family history.

**Fig. 1:**
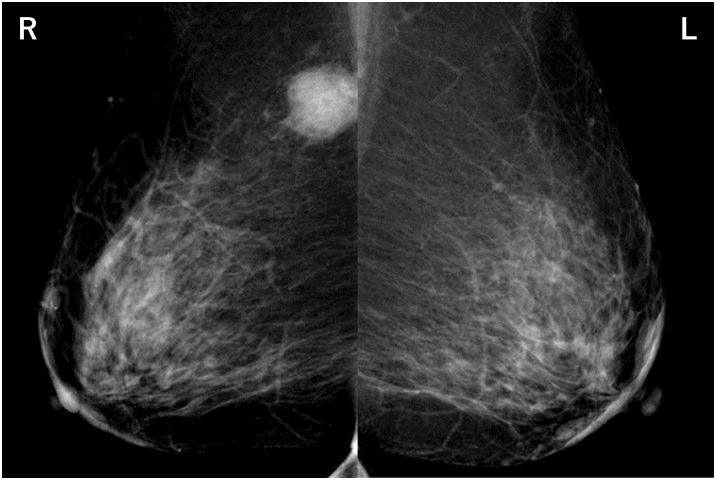
Mammographic findings. A high-density mass with a spiculated margin was observed in the upper breast on the mediolateral oblique view and was assessed as category 4. The left breast was assessed as category 1.

**Fig. 2:**
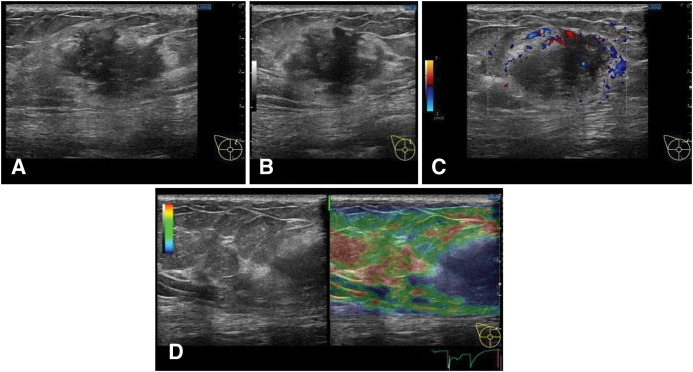
Breast ultrasonographic findings. Ultrasonography revealed an irregular, hypoechoic mass measuring 36 mm in diameter in the upper inner portion of the right breast (**A**, **B**). The mass was surrounded by a thick halo and showed increased peripheral vascularity with mild internal blood flow signals (**C**). US elastography demonstrated markedly decreased strain in the lesion, corresponding to a Tsukuba elasticity score of 5 (**D**). No axillary lymph node enlargement was observed.

**Fig. 3:**
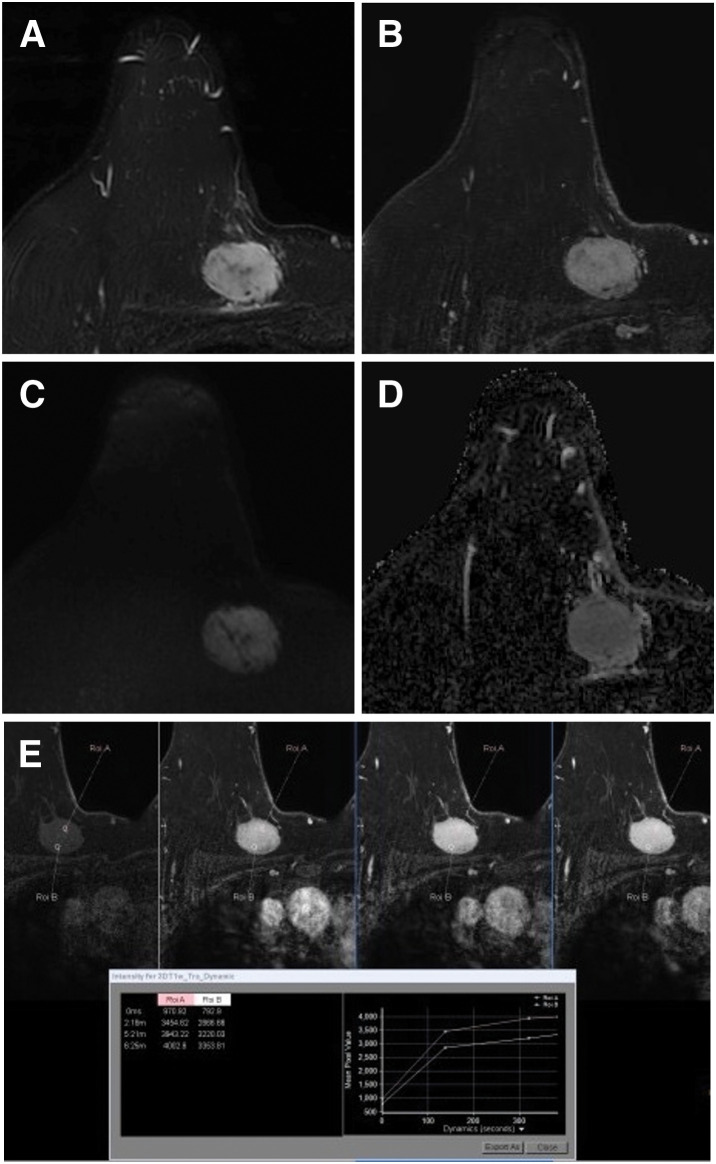
Breast MRI findings. (**A**) An oval mass with irregular margins showed high signal intensity on fat-suppressed T2-weighted imaging and (**B**) mild, relatively homogeneous enhancement on early-phase contrast-enhanced T1-weighted imaging. (**C**) Diffusion-weighted imaging and (**D**) the apparent diffusion coefficient map show no definite diffusion restriction. (**E**) The time–signal intensity curve showed a fast–persistent enhancement pattern.

On physical examination, a well-mobile mass measuring approximately 3 cm in diameter was palpated in the upper inner quadrant of the right breast, slightly toward the cranial side. No skin thickening or inflammatory changes were observed. No axillary lymphadenopathy was palpable. Mammography demonstrated a 3-cm high-density mass with spiculated margins in the upper inner region of the right breast (**Fig. 1**). Ultrasonography revealed an irregular mass measuring 36 × 35 mm along the medial margin of the upper right breast (**Fig. 2**). The lesion was predominantly hypoechoic and surrounded by a thick halo. Increased vascularity was observed at the periphery of the mass, with slight penetrating blood flow signals within the lesion. Elastography demonstrated marked stiffness within the lesion. No significant lymph node enlargement, including in the axilla, was detected. On contrast-enhanced breast MRI, an oval mass with irregular margins and relatively homogeneous internal enhancement was identified in the early phase of contrast-enhanced T1-weighted imaging (**Fig. 3**). The time–signal intensity curve demonstrated a fast–persistent pattern. The lesion showed high signal intensity on fat-suppressed T2-weighted imaging and low signal intensity on T1-weighted imaging, with no definite diffusion restriction. The lesion appeared localized.

**Fig. 4:**
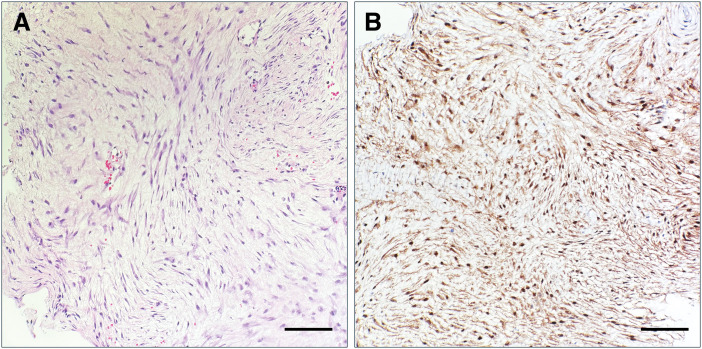
Histopathological findings of the core needle biopsy specimen. (**A**) Spindle-shaped cells with minimal cytological atypia proliferate in a fascicular pattern. (**B**) β-catenin is positive in the nuclei of the tumor cells. Scale bars represent 100 μm.

Because the imaging findings raised suspicion for breast carcinoma, a core needle biopsy was performed. The histological findings of the biopsy specimen are shown in **Fig. 4**. Microscopically, spindle-shaped cells with minimal cytologic atypia proliferated in an interlacing pattern. Immunohistochemical staining demonstrated nuclear accumulation of β-catenin, findings suggestive of desmoid-type fibromatosis. As malignancy could not be definitively excluded based on imaging, a repeat core needle biopsy was performed; however, the histological findings were similar to those of the initial biopsy, and no malignant features were identified. Although the repeated biopsy findings were consistent with desmoid-type fibromatosis, the carcinoma-like imaging features and rarity of this entity made it difficult to completely exclude malignancy based on biopsy findings alone. Therefore, surgical excision was planned as a combined diagnostic and local-control procedure for the palpable, localized lesion. After preoperative ultrasonographic marking, right breast tumor excision was performed under local anesthesia with an intended 10-mm margin, including surrounding adipose and normal breast tissue. Intraoperatively, the tumor was palpated as a firm mass, and no obvious adhesion to the surrounding tissues was observed. The superficial and deep margins were assessed by palpation, and the tumor was grossly excised with sufficient surrounding adipose tissue.

**Fig. 5:**
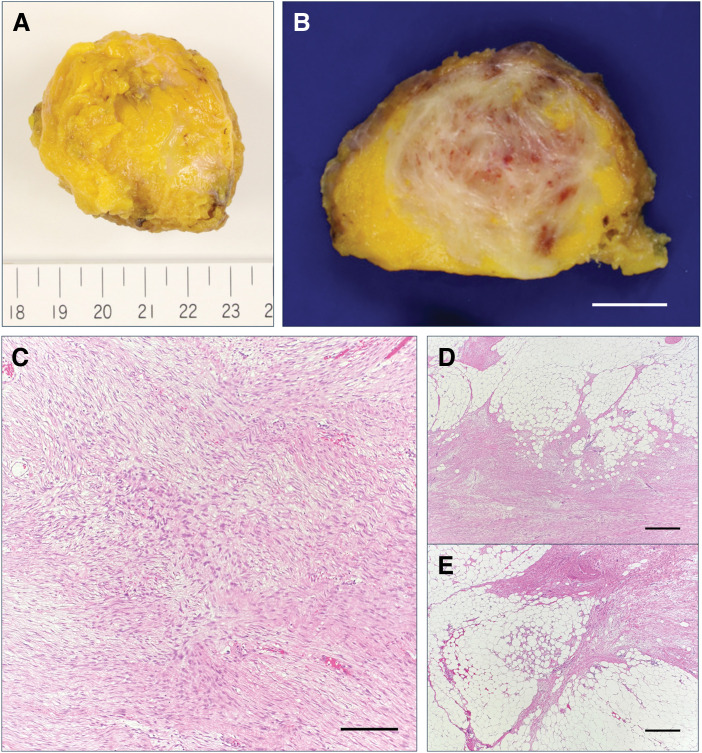
Histopathological findings of the surgically resected specimen. (**A**) The gross appearance after formalin fixation. (**B**) The cut surface revealed a yellowish-white solid lesion. (**C**) Histologically, proliferation of spindle-shaped cells was observed within the breast tissue, and nuclear atypia and mitotic figures were rarely seen. (**D**, **E**) An infiltrative growth pattern extending into the surrounding adipose tissue was observed. Scale bars represent 10 mm in (**B**), 200 μm in (**C**), and 500 μm in (**D**) and (**E**).

The postoperative pathological findings are shown in **Fig. 5**. On gross examination, the cut surface revealed a poorly demarcated, solid, yellowish-white lesion measuring 40 mm in greatest dimension. Histologically, spindle cell proliferation was observed within the breast parenchyma, showing an infiltrative growth pattern extending into the surrounding adipose tissue. No significant nuclear atypia or mitotic activity was observed. The final diagnosis was desmoid-type fibromatosis. Microscopic examination revealed focal involvement of the resection margin. Positive margins were observed mainly on the superficial side at multiple sites, with a maximum extent of 4 mm (**Supplementary Fig. 1**).

Although the final pathological diagnosis demonstrated focal positive margins, the patient opted for observation rather than additional excision after discussion. At 3 months after surgery, no evidence of recurrence was observed. Postoperative follow-up is planned with ultrasonography every 6 months.

## DISCUSSION

Desmoid-type fibromatosis arising in the breast is a rare tumor, and only a limited number of cases have been reported. The age at onset is not restricted to a specific age group.^6)^ Although associations with hormonal factors, prior breast surgery, and trauma have been suggested, the precise pathogenesis remains unclear.^6,7)^ Clinically, it is most often detected as a painless breast mass and may be accompanied by skin dimpling or breast deformity. On imaging, it frequently appears as a spiculated mass, making differentiation from breast carcinoma challenging.^8,9)^ Although the clinical course is generally slow-growing, the tumor is characterized by marked local infiltrative behavior and a risk of local recurrence even after surgical excision.^6)^

The most distinctive diagnostic feature of this disease is the difficulty in distinguishing it from breast carcinoma based on imaging findings. When arising in the breast, it may appear on mammography as a high-density, irregular mass with spiculated margins. On ultrasonography, it can appear as an irregular or ill-defined hypoechoic mass, making differentiation from breast cancer challenging in some cases.^4,8–10)^ Early enhancement on MRI has also been reported.^5,11)^ Therefore, histopathological examination is indispensable for a definitive diagnosis.^4,8)^ These imaging findings reflect the pathological characteristics of desmoid-type fibromatosis, which is characterized by the proliferation of fibrous stroma with infiltrative extension into the surrounding tissue. As a result, the lesion may demonstrate ill-defined, irregular, and spiculated margins on imaging, closely mimicking a malignant tumor. The imaging findings in the present case were consistent with the known pathological features of desmoid-type fibromatosis. Histological examination of the surgical specimen revealed infiltrative growth into the surrounding adipose tissue, corresponding to the imaging findings. However, given the rarity of this disease, it was difficult to conclude preoperatively that the lesion was benign based solely on imaging features.

In a systematic review and meta-analysis of desmoid-type fibromatosis overall, which integrated data from 1295 extra-abdominal cases, microscopic positive margins were shown to be significantly associated with an increased risk of local recurrence (relative risk 1.78, 95% CI 1.40–2.26),^12)^ indicating that margin status is an important factor related to recurrence. In cases of primary breast involvement, Wargotz et al. analyzed 28 cases of mammary fibromatosis not involving the deep fascia or chest wall and reported that 5 of 20 patients (25%) who underwent local excision experienced recurrence; all recurrent cases had positive margins at the initial surgery.^13)^ Furthermore, Neuman et al., in a 25-year review of 32 cases of breast desmoid tumors, reported that 8 of 28 patients (29%) with available follow-up developed recurrence. Recurrence occurred in 5 of 9 patients (56%) with positive margins and in 3 of 19 patients (16%) with negative margins.^14)^ Although margin status alone cannot completely predict recurrence, these findings suggest that positive margins are associated with a higher recurrence rate even in primary breast cases and that securing adequate surgical margins is important. Despite preoperative image-guided marking and excision with an intended margin in the present case, microscopic involvement of the superficial margin was identified at multiple foci, illustrating the difficulty of achieving pathologically negative margins in this infiltrative tumor. Additional excision was not performed in accordance with the patient’s preference. Although data specifically describing the timing of recurrence in breast desmoid tumors are limited, studies of extra-abdominal desmoid tumors suggest that most recurrences occur within 3 years after treatment and nearly all within 6 years.^15)^ Therefore, careful long-term follow-up, potentially for at least 5 years, is considered necessary.

In recent years, the treatment strategy for desmoid-type fibromatosis has undergone a substantial shift. Although surgical resection was previously considered the first-line treatment, the recognition of cases demonstrating spontaneous regression or long-term stability has led to a change in management. Current international consensus guidelines recommend active surveillance as the initial approach for asymptomatic patients or those without functional impairment.^16,17)^ However, these guidelines are not specific to breast lesions, and evidence for breast desmoid-type fibromatosis remains limited. Kangas-Dick et al. proposed that active surveillance may be considered for breast lesions that are minimally symptomatic and/or minimally disfiguring.^3)^ Therefore, treatment decisions for breast lesions should consider diagnostic certainty, tumor growth, symptoms, local-control needs, and cosmetic outcomes, rather than applying active surveillance uniformly. In the present case, the lesion was clinically palpable and highly suspicious for breast carcinoma on imaging; therefore, surgical excision was selected as a combined diagnostic and local-control procedure. However, if the lesion had been non-palpable and small, it might have been reasonable to consider careful observation without immediate surgical resection once the diagnosis of desmoid-type fibromatosis was established by biopsy. Accordingly, it is important for clinicians to recognize the imaging characteristics of this disease and the recent paradigm shift in its management, and to guide optimal treatment decisions based on an appropriate histopathological diagnosis.

## CONCLUSIONS

Desmoid-type fibromatosis of the breast is a rare entity that can mimic breast carcinoma on imaging. Accurate histopathological diagnosis is essential for appropriate management. While surgical excision may be required in cases with strong clinical suspicion of malignancy, active surveillance represents a valid alternative for small, non-palpable lesions once the diagnosis is confirmed, reflecting the recent shift in treatment strategy.

## SUPPLEMENTARY MATERIALS

Supplementary Figure 1 Pathological grossing diagram (A) The pathological grossing diagram is shown. The light blue linesindicate the area where the tumor was exposed.
(B) The exposed area measuring 4 mm on the superficial side in specimen #7 is shown (arrows). The horizontal scale bar represents 1 mm.

## References

[1] WHO Classification of Tumours Editorial Board.Desmoid fibromatosis. In: WHO classification of tumours: soft tissue and bone tumours, 5th ed. Lyon: International Agency for Research on Cancer; 2020: 93–95.

[2] Honma N, Yoshida M, Kinowaki K, et al. The Japanese breast cancer society clinical practice guidelines for pathological diagnosis of breast cancer, 2022 edition. Breast Cancer 2024; 31: 8–15. 37934318 10.1007/s12282-023-01518-6 PMC10764572

[3] Kangas-Dick A, Ali M, Poss M, et al. Diagnosis and management of desmoid fibromatosis of the breast. World J Oncol 2024; 15: 394–404. 38751692 10.14740/wjon1844 PMC11092408

[4] Glazebrook KN, Reynolds CA. Mammary fibromatosis. AJR Am J Roentgenol 2009; 193: 856–60. 19696302 10.2214/AJR.08.1892

[5] Nakazono T, Satoh T, Hamamoto T, et al. Dynamic MRI of fibromatosis of the breast. AJR Am J Roentgenol 2003; 181: 1718–9. 14627606 10.2214/ajr.181.6.1811718

[6] Devouassoux-Shisheboran M, Schammel MD, Man YG, et al. Fibromatosis of the breast: age-correlated morphofunctional features of 33 cases. Arch Pathol Lab Med 2000; 124: 276–80. 10656738 10.5858/2000-124-0276-FOTB

[7] Bouab M, Harit A, Boufettal H, et al. Desmoid fibromatosis of the breast occurring after breast reduction surgery mimicking a carcinoma: a rare case report. Ann Med Surg (Lond) 2022; 77: 103526. 35638040 10.1016/j.amsu.2022.103526 PMC9142379

[8] Ebrahim L, Parry J, Taylor DB. Fibromatosis of the breast: a pictorial review of the imaging and histopathology findings. Clin Radiol 2014; 69: 1077–83. 24990452 10.1016/j.crad.2014.05.105

[9] Taliaferro AS, Fein-Zachary V, Venkataraman S, et al. Imaging features of spindle cell breast lesions. AJR Am J Roentgenol 2017; 209: 454–64. 28537752 10.2214/AJR.16.17610

[10] Wuyts L, De Schepper A. Desmoid-type fibromatosis of the breast mimicking carcinoma. J Belg Soc Radiol 2019; 103: 13. 30706051 10.5334/jbsr.1612 PMC6354018

[11] Ng WL, Teoh SY, See MH, et al. Desmoid type fibromatosis of the breast masquerading as breast carcinoma: value of dynamic magnetic resonance imaging and its correlation. Eur J Breast Health 2021; 17: 197–9. 33870121 10.4274/ejbh.galenos.2020.5482 PMC8025715

[12] Janssen ML, van Broekhoven DL, Cates JM, et al. Meta-analysis of the influence of surgical margin and adjuvant radiotherapy on local recurrence after resection of sporadic desmoid-type fibromatosis. Br J Surg 2017; 104: 347–57. 28199014 10.1002/bjs.10477

[13] Wargotz ES, Norris HJ, Austin RM, et al. Fibromatosis of the breast: a clinical and pathological study of 28 cases. Am J Surg Pathol 1987; 11: 38–45. 10.1097/00000478-198701000-00005 3789257

[14] Neuman HB, Brogi E, Ebrahim A, et al. Desmoid tumors (fibromatoses) of the breast: a 25-year experience. Ann Surg Oncol 2008; 15: 274–80. 17896146 10.1245/s10434-007-9580-8

[15] Papagelopoulos PJ, Mavrogenis AF, Mitsiokapa EA, et al. Current trends in the management of extra-abdominal desmoid tumours. World J Surg Oncol 2006; 4: 21. 16584569 10.1186/1477-7819-4-21 PMC1456964

[16] Alman B, Attia S, Baumgarten C, et al. The management of desmoid tumours: a joint global consensus-based guideline approach for adult and paediatric patients. Eur J Cancer 2020; 127: 96–107. 32004793 10.1016/j.ejca.2019.11.013

[17] Mangla A, Agarwal N, Schwartz G. Desmoid tumors: current perspective and treatment. Curr Treat Options Oncol 2024; 25: 161–75. 38270798 10.1007/s11864-024-01177-5 PMC10873447

