# Supplementary material for: Breast Desmoid-Type Fibromatosis Mimicking Carcinoma on Imaging: A Case Report
Source: Surg Case Rep. 2026 Jun 13;12(1):26-0213. doi: 10.70352/scrj.cr.26-0213 (PMC13271897; doi:10.70352/scrj.cr.26-0213)
Supplement: Supplementary Figure 1 — (A) The pathological grossing diagram is shown. The light blue linesindicate the area where the tumor was exposed. (B) The exposed area measuring 4 mm on the superficial side in specimen #7 is shown (arrows). The horizontal scale bar represents 1 mm. [file scr-12-01-26-0213-s001.pdf]

Supplementary Figure 1. Pathological grossing diagram

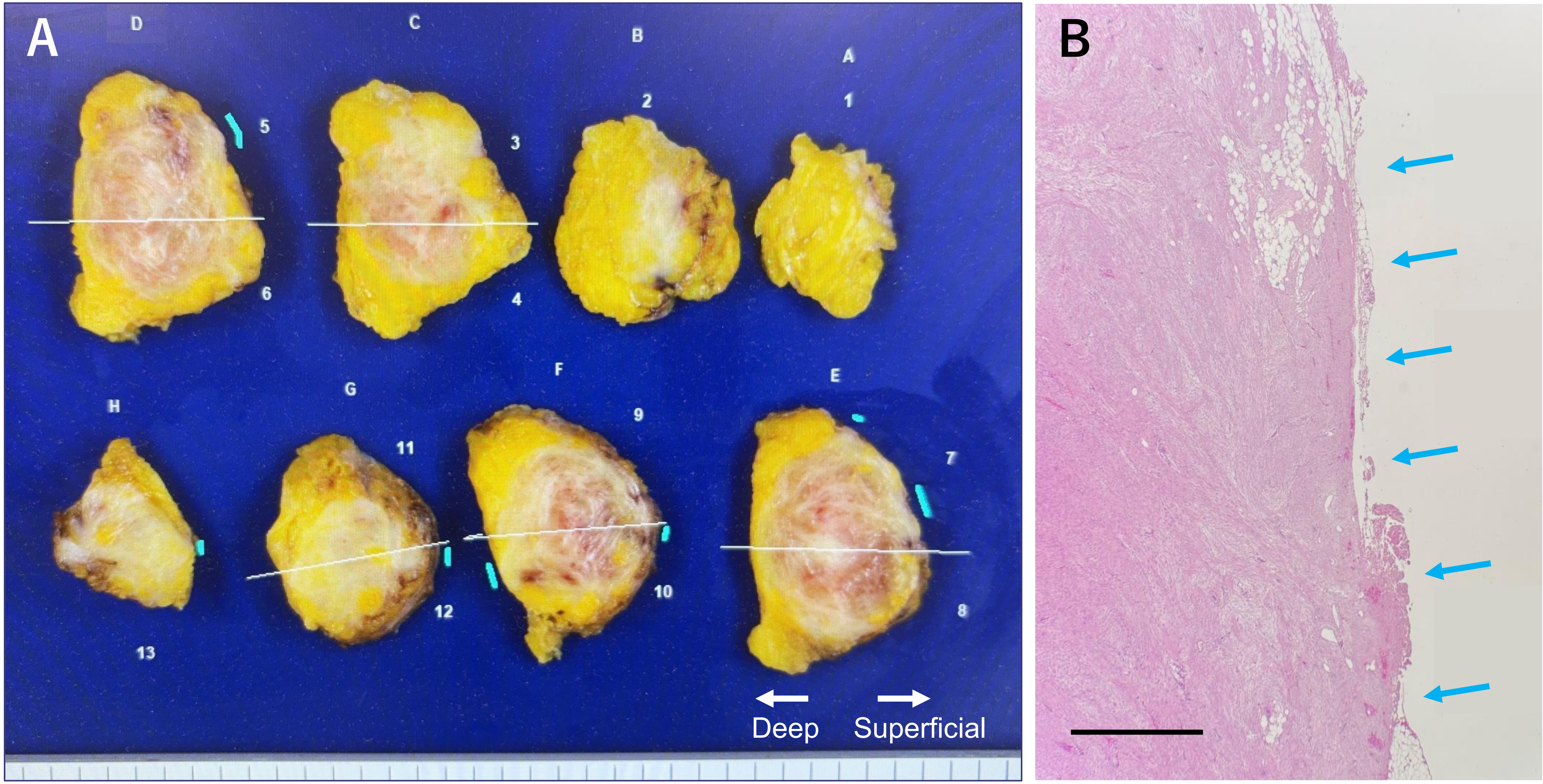

(A) The pathological grossing diagram is shown. The light blue lines indicate the area where the tumor was exposed.
(B) The exposed area measuring 4 mm on the superficial side in specimen #7 is shown (arrows). The horizontal scale bar represents 1 mm.
